# Altered Regional Brain Cortical Thickness in Pediatric Obstructive Sleep Apnea

**DOI:** 10.3389/fneur.2018.00004

**Published:** 2018-01-22

**Authors:** Paul M. Macey, Leila Kheirandish-Gozal, Janani P. Prasad, Richard A. Ma, Rajesh Kumar, Mona F. Philby, David Gozal

**Affiliations:** ^1^School of Nursing, University of California, Los Angeles, CA, United States; ^2^Brain Research Institute, University of California, Los Angeles, CA, United States; ^3^Department of Pediatrics, Section of Pediatric Sleep Medicine, Pritzker School of Medicine, Biological Sciences Division, The University of Chicago, Chicago, IL, United States; ^4^Department of Anesthesiology, University of California, Los Angeles, CA, United States; ^5^Department of Radiological Sciences, David Geffen School of Medicine at UCLA, University of California, Los Angeles, CA, United States

**Keywords:** sleep disordered breathing, cortex, intermittent hypoxia, atrophy, obstructive sleep apnea, cognitive deficits

## Abstract

**Rationale:**

Obstructive sleep apnea (OSA) affects 2–5% of all children and is associated with cognitive and behavioral deficits, resulting in poor school performance. These psychological deficits may arise from brain injury, as seen in preliminary findings of lower gray matter volume among pediatric OSA patients. However, the psychological deficits in OSA are closely related to functions in the cortex, and such brain areas have not been specifically assessed. The objective was to determine whether cortical thickness, a marker of possible brain injury, is altered in children with OSA.

**Methods:**

We examined regional brain cortical thicknesses using high-resolution T1-weighted magnetic resonance images in 16 pediatric OSA patients (8 males; mean age ± SD = 8.4 ± 1.2 years; mean apnea/hypopnea index ± SD = 11 ± 6 events/h) and 138 controls (8.3 ± 1.1 years; 62 male; 138 subjects from the NIH Pediatric MRI database) to identify cortical thickness differences in pediatric OSA subjects.

**Results:**

Cortical thinning occurred in multiple regions including the superior frontal, ventral medial prefrontal, and superior parietal cortices. The left side showed greater thinning in the superior frontal cortex. Cortical thickening was observed in bilateral precentral gyrus, mid-to-posterior insular cortices, and left central gyrus, as well as right anterior insula cortex.

**Conclusion:**

Changes in cortical thickness are present in children with OSA and likely indicate disruption to neural developmental processes, including maturational patterns of cortical volume increases and synaptic pruning. Regions with thicker cortices may reflect inflammation or astrocyte activation. Both the thinning and thickening associated with OSA in children may contribute to the cognitive and behavioral dysfunction frequently found in the condition.

## Introduction

Pediatric obstructive sleep apnea (OSA) has a prevalence of up to 5% and is the leading sleep disorder in children (1, 2). The condition is characterized by repeated intermittent upper airway obstruction during sleep, which results in episodic alveolar hypoventilation and hypoxemia, as well as sleep fragmentation (2). The condition leads to multiple daytime symptoms and is associated with an increased risk for severity-dependent cognitive, behavioral, and cardiovascular and metabolic morbidities, all of which likely impact optimal behavioral and health development (3–11). In addition, the cognitive and behavioral deficits frequently encountered in pediatric OSA likely lead to poor school performance (12–15). Brain injury is a possible mechanism underlying these symptoms, as shown in the recently reported gray matter changes in pediatric OSA (16). These earlier findings showed general regions with lower gray matter volume, but many structures of specific interest to cognitive function and school performance were not assessed. In particular, the methodology was suited to detecting changes deep in the brain, whereas much executive function and cognitive processing is centered in cortical structures. This lack of understanding of whether and how cortical regions are affected limits selection of possible interventional targets, particularly for the cognitive difficulties, which are especially important for children in relation to their scholastic development.

Previous studies examining OSA in adults have shown brain injury and physiological and psychological symptoms similar to those detected in pediatric OSA. Neuroimaging studies in OSA report alterations of white and gray matter, as well as changes in neurochemicals within various brain regions, raising the possibility that pediatric patients may also exhibit such brain changes (17–22). Our earlier study in children with OSA focused on regional gray matter volume assessment showed selected limbic, subcortical, and brainstem regions with reduced gray matter (16). These brain regions, along with other sites, control cognition and mood, such that OSA-induced injury may underlie the cognitive deficits frequently seen in pediatric OSA. Given that more frequent behavioral symptoms are seen in even milder forms of OSA (23, 24), more widespread cortical areas may be impacted. However, the “voxel-based morphometry” technique used for the earlier gray matter quantitation is less sensitive to changes in outer cortical regions that in deeper brains structures, thus raising the need to examine regional cortical sites with a different approach.

Cortical thickness is one measure that is highly sensitive to changes only in neuronal cortical areas (25), and both the cognitive and behavioral findings suggest that cortex-specific regions may be affected, perhaps due to injury from the intermittent hypoxia or sleep fragmentation in pediatric OSA (7, 11, 26). Cortical thickness examination can be performed with standard image processing techniques, such as FreeSurfer software (27). This approach involves detecting the inner and outer surfaces of the cortical gray matter, and calculating the thickness as the distance between the two surfaces. Specialized statistics are used to assess thinning or thickening of the cortex, and this methodology has been applied to many conditions.

The purpose of this study was to assess potential injury to the brain cortical regions in children diagnosed with OSA using polysomnography. Building on earlier findings of gray matter injury in mixed cortical and subcortical areas (16), we hypothesized that regional cortical thickness alterations would occur in many areas in pediatric OSA subjects, and more specifically in those regions that are involved in cognition.

## Materials and Methods

### Subjects

The study was approved by the human ethics committee at the University of Chicago (IRB Protocol # 11-0280-CR004) and written informed consent was obtained from the legal caregiver of each participant in accordance with the Declaration of Helsinki. Assent was obtained from children >7 years old. Children being evaluated for habitual snoring, who were diagnosed as OSA with an overnight polysomnography, were invited to participate. Invitations were given consecutively to children who visited the clinic until the sample was attained. Controls were recruited through our well-child clinics, after ascertaining that they did not snore based on a validated questionnaire, and further subjected to a sleep study to confirm the absence of any evidence indicating either snoring or more severe forms of sleep-disordered breathing. Participants underwent baseline anthropometric assessments, as well as overnight polysomnography, which were interpreted using standard criteria (28, 29). In addition, a neurocognitive battery was also administered to all participants in the morning (starting at 9:00 a.m.) after breakfast and following the sleep study. The details and specific tests included in the cognitive battery have been previously reported in great detail (11). In brief, the cognitive tests administered the morning following polysomnographic assessment consisted of the Differential Ability Scales (DAS) (30). This battery of tests has been developed and standardized for assessment of aspects of broad cognitive functioning, as well as more specific domains of neuropsychological status, and is frequently utilized diagnostically in educational and clinical settings. Results are normalized to a standardized score of 100 with a SD of 10 for general conceptual ability, which is viewed as a surrogate of intellectual quotient. All methods were performed in accordance with the relevant guidelines and regulations.

### Exclusion Criteria

Children were excluded from the study if they were diagnosed with ADHD, were using psychostimulant medications (*n* = 5), or exhibited known neurodevelopmental delays (*n* = 1). In addition, children with hypertension or using antihypertensive drug therapies were excluded (*n* = 2). Furthermore, children with either known or suspected diabetes, as delineated by the Global IDF/ISPAD Guideline for Diabetes in Childhood and Adolescence^1^ (*n* = 1), with a craniofacial, neuromuscular, or defined genetic syndrome, and children on chronic anti-inflammatory therapy (*n* = 1), or with any known acute or chronic illness were also excluded. MRI scanning exclusion criteria included metallic implants and claustrophobia. MRI data exclusion criteria include motion and other artifacts.

### Anthropometry

The Centre for Disease Control 2000 and The Children’s Hospital of Philadelphia online software^2^ were used to calculate height and weight centiles and body mass index (BMI) *Z*-scores respectively. Obesity was defined as BMI *Z*-scores >1.65.

### Sphygmomanometry

All children’s arterial blood pressure was measured. Using National Heart, Lung, and Blood Institute guidelines,^3^ systolic and diastolic BP indices (SBPi and DBPi, respectively) were calculated by dividing the average systolic and diastolic pressure by the respective 95th percentile for BP, computed for age, sex, and height. SBPi or DBPi pressures >1 was categorized as hypertension.

### Overnight Polysomnography

As previously described, standard approaches were used to conduct overnight polysomnography (28, 29). The criteria for OSA diagnosis consisted of an obstructive apnea/hypopnea index (AHI) >2/h total sleep time (TST) and a nadir SpO2 <92%, and/or a respiratory arousal index >2/h TST (31, 32).

### Neurocognitive Assessments

The cognitive tests were conducted in the morning after the sleep study and consisted of the DAS (30). The school age form of the DAS was administered, which yields a Spatial Cluster score in addition to the verbal, nonverbal, and global composite score, the latter called the general conceptual ability (GCA) score. The sum of the core subtest *t*-scores is converted to a total battery standard score, the GCA, with a mean of 100 and a SD of 15 (33).

### MRI Scanning

Some control and all OSA subjects were scanned at The University of Chicago, and a large number of healthy subjects from the NIH Pediatric MRI database were used to supplement the control group.

We collected brain MRI data from children within 3–5 days after the sleep study on a Philips Achieva 1.5-T scanner. High-resolution three-dimensional T1-weighted anatomical scans were collected from 16 OSA and 9 control subjects using a custom ultrafast gradient echo “SENSE” sequence (repetition time = 8.16 ms; TE = 3.7 ms; flip angle = 8°; matrix size = 256 × 256; field of view = 224 mm × 224 mm; slice thickness = 1.0 mm; number of slices = 160). Six additional control subjects were originally scanned, but were excluded from analysis due to motion artifacts in the images.

An additional 138 control subjects were included from a national database. We downloaded high-resolution T1-weighted images of remaining control subjects from the NIH Pediatric MRI database^4^ with permission. We used a large control cohort, since increasing cohort size is one approach to improve reliability of volume-based analyses in OSA (34). Note that this “population control” group cannot be assumed to have identical characteristics as the nine subjects studied at Chicago, which would affect any sample size power or sensitivity calculations. Full details of recruitment and scanning protocols are available from the project website, including confirmation that consent and assent was obtain as appropriate for the age of the subjects, and that procedures were approved by applicable institutional review boards. In brief, the purpose of the pediatric MRI study of normal brain development includes providing a normative database of the developing brain for comparison with neuroimaging studies of pediatric disease conditions. Participants were recruited from six sites across the United States, and evaluated and screened for health status based on extensive criteria. MRI scans were acquired according to standard protocols, including high-resolution T1-weighted scans at 1 mm isotropic voxel resolution, as used in the present study. Some participants were studied at one or two follow-up visits to obtain longitudinal data. We selected 138 subjects from the database with T1-weighted scans in the age range of our OSA participants. For subjects who fell within the age range during more than one visit, only one recording was used; the choice of which visit was based on ensuring the best age match with the OSA group.

### Analysis

Demographic and sleep variables for the Chicago data were analyzed with independent samples *t*-tests and Chi-square tests.

For brain image assessments, we used the FreeSurfer and MATLAB-based SPM12 software packages for data processing and analysis. SPM12 was used to process the T1-weighted scans, including removal of signal intensity variations due to field inhomogeneities (35). The processed images were imported into FreeSurfer to assess cortical thickness (27). The initial skull stripping and boundary identification were performed, and skull strips of all subjects were manually checked to ensure no brain area was excluded. Similarly, the pial and gray–white matter boundaries were visually checked and, if needed, edits were made to correct misidentified regions. Such edits include correcting misaligned boundaries such as occasions when the automatic calculation would erroneously identify the “gray matter” edge as in the skull region. Minor edits were required to the automatically detected pial boundaries in most subjects, but the skull strip and white matter boundaries did not require any adjustment.

The FreeSurfer processing stream was followed to generate cortical thickness across the brain, except for cerebellar areas. Cortical thickness was calculated as the distance between gray and white matter surfaces. Thinning is considered to reflect atrophy or neurodegenerative processes (or underdevelopment in children), and thickening can reflect swelling due to inflammation or higher-than normal usage. Surface statistics were implemented to assess cortical thinning or thickening in OSA over control subjects, using a general linear model with group and sex as independent variables. Sex was included to account for possible sex differences in brain structure, but was treated as a covariate of no interest, and was not assessed for effects (“nuisance” variable in FreeSurfer terminology). We also assessed correlations of cortical thickness with DAS scores in participants who had these measures. Each hemisphere was analyzed separately, with 10 mm smoothing. We used a threshold of *P* ≤ 0.05 with false discovery rate correction (FDR) for multiple comparisons. We overlaid the areas of significant difference onto the inflated cortical surface (sulcal and gyral areas displayed as smooth adjacent regions without depth).

Findings show regions of altered cortical thickness. Such regions are termed “clusters” in reference to the multiple adjacent surface points that comprise the region. A cluster is defined as a group of surface points showing a significant thickening or thinning. Clusters are displayed visually, and quantitative measures of mean thickness and statistical differences are reported for each cluster (reporting by individual surface location would result in excessive numerical values.). The FreeSurfer software matches the locations of clusters to a standard atlas, providing standard labels of the regions affected; these labels are indicated in the results.

## Results

Sixteen children with OSA and nine age, sex, ethnicity, and BMI *Z*-score matched controls underwent sleep studies, neurocognitive testing, and MR imaging. Demographic and polysomnography data for those subjects are listed in Table 1, showing any pertinent differences in sleep characteristics and BMI *Z* scores.

**Table 1:** Polysomnographic and demographic data of 16 obstructive sleep apnea (OSA) and 9 control subjects studied at the University of Chicago.

	OSA (*n* = 16)	Control (*n* = 9)	*P* (chi-square or *t*-test)
Age (years)	8.4 ± 1.2	9.2 ± 1.7	0.18
Gender	8 females, 8 males	4 females, 5 males	0.77
Ethnicity	8 African American, 7 caucasian, 1 other	5 African American, 4 caucasian	0.78
Body mass index *Z* score	1.3 ± 0.2	1.1 ± 0.2	0.02*
Systolic BP (mmHg)	108 ± 10	102 ± 8	0.14
Diastolic BP (mmHg)	67 ± 7	63 ± 7	0.18
AHI (events/h)	11 ± 6	0.4 ± 0.2	<0.001*
S_p_O_2_ Nadir (%)	77 ± 12	94 ± 3	<0.001*
ODI 3% (/hrTST)	12 ± 11	0.2 ± 0.2	<0.001*
Total arousal index (/hrTST)	17 ± 5	7.3 ± 2.8	<0.001*
DAS general conceptual ability score	89.6 ± 8.0	103.8 ± 3.2	<0.001*
Median/interquartile range	88 (11.5)	105 (4)	

*Continuous variables are mean ± SD*.

*DAS, differential ability scales; hrTST, hours of total sleep time; ODI 3%, 3% oxygen desaturation index; AHI, apnea/hypopnea index*.

*P ≤ 0.05.

**Figure 1:**
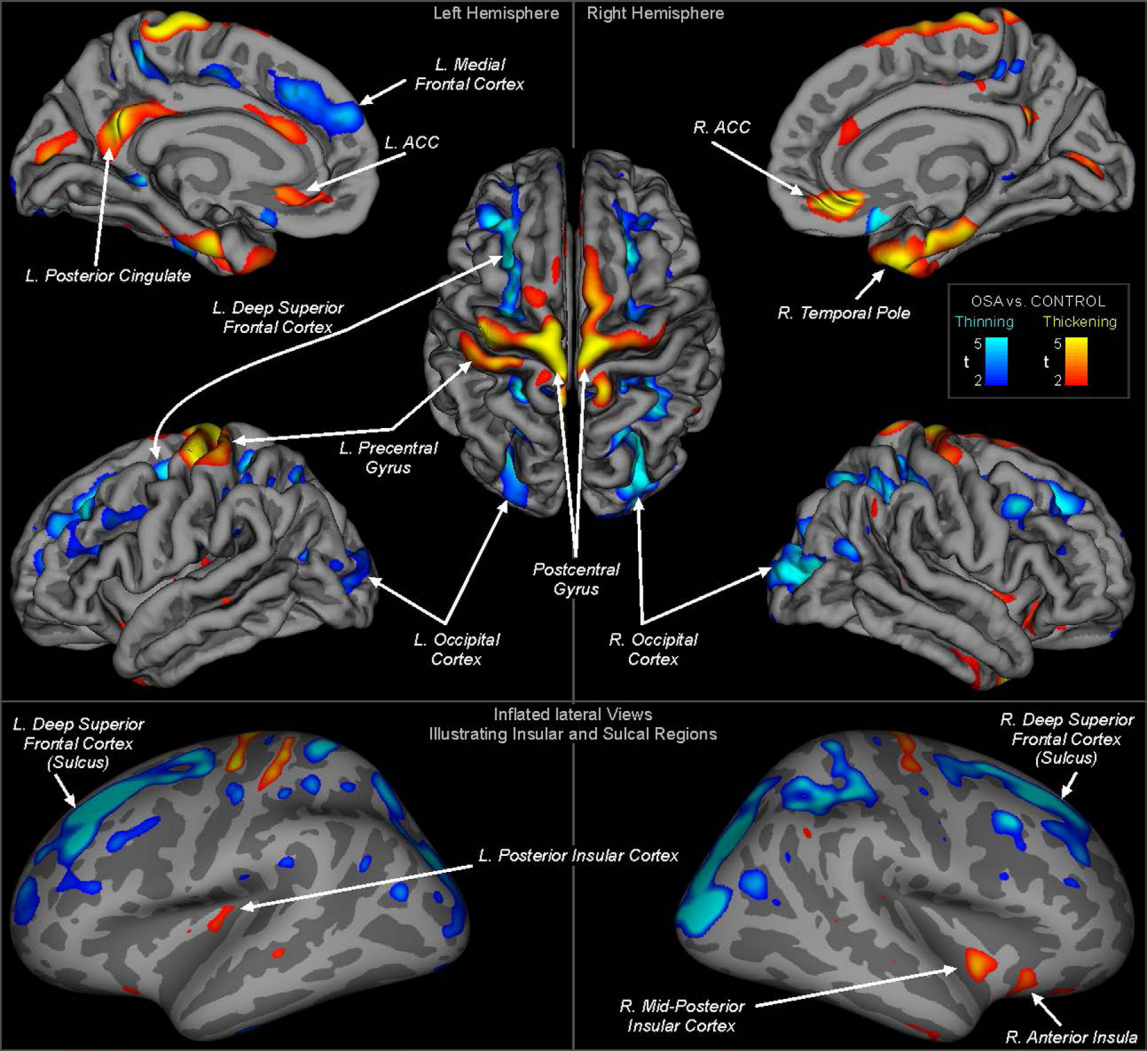
Regions of significant cortical thickness differences between obstructive sleep apnea and controls (FDR ≤0.05). Areas are color-coded according to significance level (*t*-statistic), with cool colors representing thinning and warm colors thickening (see scale). The top panels show regions overlaid onto the pial surface (gray matter boundary) for clear visualization of structures and gyral differences. The lower panel shows regions overlaid onto an inflated view, whereby all regions including sulci and the insular cortices are visible. Light gray shading represents gyral and dark gray sulci. ACC, anterior cingulate cortex. Quantitative measures for each region are shown in Tables 2 and 3.

Whole-brain group comparisons of the 16 OSA patients with a control group consisting of the nine controls and the 138 Pediatric MRI subjects revealed cortical thinning associated with OSA in various areas including the superior and medial frontal, prefrontal and parietal cortices, and the occipital cortex (FDR = 0.05). Regions of significantly thicker or thinner cortex in OSA are shown in Table 2 (left hemisphere) and Table 3 (right hemisphere). Representations of these cortical thickness differences between control and OSA groups in the left and right hemispheres are shown in Figure 1 from multiple views overlaid onto the FreeSurfer template pial surface, and a lateral, inflated view to illustrate otherwise hidden structures (i.e., insular cortex, and sulci).

**Table 2:** Left hemisphere clusters of significant cortical thickness differences between obstructive sleep apnea and controls [false discovery rate (FDR)] ≤0.05, corresponding to *t* = 2.15].

Max *t*-statistic	Size (mm^2^)	*X*	*Y*	*Z*	*N* vertices	Region by cortical label (applies to cortex, gyrus, or lobe)
**Thinning**						

−10.8	2,709.27	−28.1	13.1	42.4	4,948	Caudal middle frontal/superior frontal, rostral middle frontal, superior frontal
−6.187	325.93	−22.3	−46.6	58.3	718	Superior parietal
−5.5355	1,771.05	−25.6	−77.4	15.2	3,046	Superior parietal/lateral occipital
−4.8206	67.77	−12.1	−42.6	−0.9	195	Isthmus cingulated
−4.7227	175.37	−47.3	−19.8	−31.3	283	Inferior temporal
−4.4787	732.12	−8	52.1	28.1	1,128	Superior frontal
−4.3941	344.78	−17.1	−42.4	55.8	921	Paracentral
−4.3517	187.31	−8.1	−8.6	50.5	462	Superior frontal
−4.0071	125.96	−32.6	−42.5	47.8	281	Superior parietal
−3.9568	447.87	−24.1	48.3	13.3	689	Rostral middle frontal (prefrontal)
−3.7806	138.57	−38.3	−22.3	40.7	382	Postcentral
−3.774	77.37	−40	−69.6	15	169	Inferior parietal
−3.6777	52.37	−40.3	−30.6	44.7	146	Postcentral
−3.6102	42.77	−6.1	18.7	−17.2	102	Medial orbitofrontal (ventral prefrontal)
−3.3606	275.52	−42.3	21.7	31.8	425	Rostral middle frontal (prefrontal)
−3.2277	50.18	−41.6	−56.1	25	113	Inferior parietal
−3.0804	50.9	−29.4	−51.1	48.8	122	Superior parietal
−3.0155	74.9	−53.3	−35.3	26.7	127	Supramarginal
−2.7616	28.47	−19.3	−31.6	55.2	96	Precentral
−2.7278	102.78	−29.6	−89	−15.1	136	Lateral occipital
−2.6423	43.26	−36.3	−83	11	66	Inferior parietal
−2.6211	31.74	−10.5	−54.8	47.8	70	Precuneus
−2.4388	48.87	−13.8	−101.9	−3.1	69	Lateral occipital
2.416	30.05	−7.8	12.3	64.4	71	Superior frontal
−2.2875	16.18	−53.3	−19.7	21.4	38	Supramarginal
2.201	15.98	−7.7	−84	−5.2	13	Lingual
−2.1651	3.17	−12.2	54.6	26.1	5	Superior frontal
2.1619	3.13	−36.6	−9	3.3	6	Insula

**Thickening**						

9.2562	865.33	−9.1	−26	73.5	2,088	Precentral
6.9428	679.07	−10	−51.3	27.6	1,646	Isthmus cingulate/posterior cingulate, precuneus
5.1711	735.08	−21.2	−11.1	−31.4	1,571	Entorhinal/parahippocampal, temporal pole
4.5845	290.72	−33.8	−32.3	66.9	856	Postcentral
4.0226	279	−4.4	−86.9	10.1	340	Cuneus
3.6853	219.32	−6.4	22.6	21.3	488	Caudal anterior cingulate
3.5736	272.56	−5.8	22.8	−6.8	554	Rostral anterior cingulate
3.0479	41.7	−14.4	−15.9	39.9	131	Posterior cingulate
2.7609	82.39	−15.8	−1.8	68.5	173	Superior medial frontal
2.7575	118.89	−34.4	−22.3	0.3	274	Insula
2.6461	44.82	−25.2	16.7	−17.3	105	Lateral orbitofrontal (ventral prefrontal)
2.6231	22.87	−46.8	−33.6	−6.7	71	Middle temporal
2.5621	50.04	−38.6	−47.3	−20.1	88	Fusiform
2.4851	23.56	−12.2	−38.6	75.2	66	Postcentral

*Negative *t*-values represent thinning and positive values thickening. *X*/*Y*/*Z* are Talairach coordinates in millimeters. The region is based on the FreeSurfer atlas annotation, with additional labels for larger clusters that cover more than one structure. Standard terms are indicated in brackets beside some FreeSurfer labels [e.g., “orbitofrontal (ventral prefrontal)”]. The number of vertices (“*N* vertices”) is an indication of the size of the cluster; each vertex is a point on the FreeSurfer representation of the cortical surface, with approximately 300,000 vertices across the whole brain. Regions are illustrated in Figure 1*.

**Table 3:** Right hemisphere clusters of significant cortical thickness differences between obstructive sleep apnea and controls [false discovery rate (FDR) ≤0.05, corresponding to *t* = 2.15].

Max *t*-statistic	Size (mm^2^)	*X*	*Y*	*Z*	*N* vertices	Region by cortical label (applies to cortex, gyrus, or lobe)
**Thinning**						

−10.3868	2,998.2	29.6	−81.6	5.9	4,854	Lateral occipital/superior parietal, inferior parietal
−7.9676	99.75	7.2	15	−16.4	226	Medial orbitofrontal (ventral prefrontal)
−6.8607	1,893.82	21.8	26.1	44.2	3,867	Superior frontal/middle frontal, precental
−5.81	1,703.74	33.3	−32.9	40	4,520	Supramarginal/inferior parietal
−5.6423	290.34	38.8	15.6	39.3	524	Caudal middle frontal
−4.1615	216.76	37.7	−63.3	19.3	409	Inferior parietal
−3.5852	73.03	17.4	−39.6	50	219	Paracentral
−3.2946	48.85	10.6	−24.7	48.9	114	Paracentral
−3.1729	59.15	6.3	−48.2	50.6	165	Precuneus
−3.0119	25.33	18.7	−31.2	55.4	85	Precentral
−2.7184	44.67	38	33.8	18.6	78	Rostral middle frontal (prefrontal)
−2.6278	25.11	35.4	−12.7	43.2	57	Precentral
−2.4642	22.3	42.4	11.1	20.9	42	Pars opercularis
−2.4079	27.81	12.3	57.2	−17.6	40	Frontal pole
−2.3123	20.7	13.4	−42.2	0.4	68	Isthmus cingulate
−2.2818	11.66	44.7	−56	27.8	30	Inferior parietal
−2.2442	11.22	44.2	−56.8	9.4	28	Inferior parietal
−2.2177	13.07	24.4	−98.4	−12	17	Lateral occipital
−2.1965	6.44	20.4	54.1	22	9	Rostral middle frontal (prefrontal)
−2.1875	7.09	10.8	59.7	23.7	10	Superior frontal
−2.1747	0.19	14.2	−41.1	−1.1	1	Isthmus cingulate
−2.1742	1.95	44.5	−8.3	33.1	5	Precentral
−2.1735	2.85	49.5	−5.1	26.7	7	Precentral

**Thickening**						

6.9845	1,036.92	8.5	−24.3	73.6	2,475	Precentral/superior medial frontal
6.414	1,012.9	28.9	6.2	−39	2,003	Temporal pole/parahippocampal
6.0379	482.41	7.1	35.7	−14	920	Medial orbitofrontal (ventral prefrontal)/subgenu anterior cingulate
4.9763	145.09	13.7	−42.9	74.6	365	Superior medial parietal
4.2332	214.11	36.6	−6.9	−8.9	481	Insula
4.1884	234.16	14.6	−25.7	36.6	674	Posterior cingulate
4.1183	139.25	11.2	−53.4	28.3	374	Precuneus
3.4239	360.03	14.5	−82.8	2.1	551	Pericalcarine
3.2219	143.39	29.7	24.5	−10	363	Lateral orbitofrontal (ventral prefrontal)
2.7777	68.85	21.4	32.3	−12.5	119	Lateral orbitofrontal (ventral prefrontal)
2.526	85.04	6.3	28.9	19.4	177	Caudal anterior cingulate
2.3735	23.97	54.6	−51.8	36.7	56	Inferior parietal
2.2461	9.22	10.3	10.8	35.6	23	Subgenu anterior cingulate
2.201	4.13	41.8	−34	11.5	10	Superior temporal
2.1812	1.38	8.8	−45.8	22.3	6	Isthmus cingulate
2.173	1.96	45	−38.6	5.8	7	Posterior superior temporal
2.1633	1.48	46.2	−22	−7.2	4	Superior temporal

*Negative t-values represent thinning and positive values thickening. X/Y/Z are Talairach coordinates in millimeters. The region is based on the FreeSurfer atlas annotation, with additional labels for larger clusters that cover more than one structure. Standard terms are indicated in brackets beside some FreeSurfer labels [e.g., “orbitofrontal (ventral prefrontal)”]. The number of vertices (“N vertices”) is an indication of the size of the cluster; each vertex is a point on the FreeSurfer representation of the cortical surface, with approximately 300,000 vertices across the whole brain. Regions are illustrated in Figure 1*.

There were increases in cortical thickness in the bilateral precentral gyrus, the left central gyrus, and regions in bilateral posterior-mid and right anterior insular cortices (Figure 1; Tables 2 and 3). The posterior cingulate and sub-genu of the anterior cingulate cortices, extending into medial prefrontal areas also showed increased thickness in OSA children, as did the temporal cortex and poles, the latter especially on the right side. The left side showed thickening in the precentral gyrus.

The analysis of DAS scores and cortical thickness performed in the 25 children studies at the University of Chicago did not reveal any significant relationships. The small number of subjects meant that the statistical power and hence sensitivity was low for this analysis.

## Discussion

Children with OSA exhibit evidence of significant cortical thinning in the frontal (including the superior frontal gyrus), prefrontal, parietal, temporal, and occipital cortices, all of which are consistent with the gray matter decreases previously reported (16, 36–38). Thus, the findings further attest to the presence of neural injury in pediatric OSA, extending earlier indications of injury to cortex-specific areas. These cortical regions play important roles in cognition, executive function, and memory. The frontal lobe is the central region for motor function, problem solving, memory, language, impulse control, and social behavior (39). The prefrontal cortex plays a large role in executive function, attention, and personality development (39). The prefrontal cortex continues to mature into late adolescent years and even early 1920s (40, 41), such that damage at a young age could greatly hinder the normative development processes of higher cognitive functions. Both the parietal and occipital cortices also contribute to cognition and behavior; the parietal cortex through spoken and written word comprehension, encoding, consolidating and retrieving memory, and manipulating working memory (42–44) and the occipital cortex through memory, visuoconstruction (the ability to manipulate spatial information), counting and arithmetic, and executive tasks (45–47). Therefore if damaged, as cortical thinning would suggest, the frontal, parietal, and occipital cortex could exhibit altered function, affecting neurobehavioral development and functioning.

In adult OSA, cortical thinning is often attributed to cell death or neuronal injury, as thinning constitutes the most likely imaging correlate of dramatic volume reduction in a mature brain (17, 48–51). Earlier findings of reduced gray matter volume (16) could reflect both atrophy and vascular or cell damage, including injury with atrophy, whereas cortical thinning is specific to atrophy *per se*. While direct neuronal injury is a likely event in pediatric OSA due to the intermittent hypoxia and sleep fragmentation that characterize this condition, the effect of OSA on brain maturational processes must also be taken into account. In children ages 5–11 years undergoing normal neural development, the frontal and occipital lobes increase in volume by 0.4–1.5 mm every year (52). Therefore, a disruption of neural developmental processes could be the underlying cause of volume reduction in pediatric OSA as opposed to implicitly signifying the presence of neurodegeneration. Thus, OSA could prevent normal developmental brain volume increases through the same aforementioned mechanisms implicated in neurodegeneration, resulting in thinner cortices in children with OSA than in controls.

Cortical thickening was seen in the precentral and left central gyrus, the medial prefrontal cortex, the mid and anterior insular cortex, the posterior and subgenu of the anterior cingulate cortex, and the medial temporal lobe, areas that are involved in cognitive, emotional, autonomic, pain, and motor function. The insular cortex is involved in emotional control, self-awareness and cognitive functioning and motor control (53–58). The anterior cingulate cortex also plays a role in emotions as well as reward, decision-making, and autonomic regulation including blood pressure and heart rate (59–61), while the posterior cingulate is involved in human awareness and pain and episodic memory retrieval (62–64). The medial temporal lobe is a key region for long-term memory (along with the hippocampus region) (65) and the precentral gyrus is the site of the primary motor cortex and the motor strip and controls voluntary skeletal muscle movement (66–68). Cortical alterations in these regions could result in the cognitive, autonomic, memory, and psychological symptoms experienced in OSA, including worse performance on cognitive tasks, hypertension and high sympathetic tone, which could eventually cause cardiovascular issues, memory loss, and depressive symptoms, respectively. Nonetheless, the relatively small number of OSA subjects and the fact that some children had evidence of cognitive deficits while others did not may account for the absence of a significant association between cortical thickness and cognitive test scores.

Increases in cortical thickness could also indicate abnormal neural development in pediatric OSA patients. Synaptic pruning is a normally occurring process that is actively regulated through childhood and adolescence, whereby synapses are removed in order to optimize brain function (52, 69). In children ages 5–11 years, cortical thinning occurs in the right dorsal frontal and bilateral parietals regions by about 0.15–0.30 mm per year as a result of synaptic pruning (52, 69). In pediatric OSA, the present findings suggest the normal process of synaptic pruning may be disrupted. In addition, cortical thickness may also increase due to hypoxia-induced neuro-inflammation and glial activation, indicating an immune response in affected cortical areas. Identifying the exact cause will obviously require further investigation with additional complementary imaging approaches such as diffusion tensor imaging, MR spectroscopy, or functional MRI, which would provide information on alterations in regional neurochemical substrates among pediatric OSA subjects when compared to controls.

The cognitive and behavioral symptoms reported in children with OSA are consistent with symptoms reported by sleep deprivation and disruption studies in children (70). Altered sleep duration and continuity may induce behavioral perturbations in children with sleep-disordered breathing, ultimately favoring the occurrence of dis-maturational processes in the brain and finally manifesting as poor performance in school (71). Although we found no significant associations between the structural findings and the cognitive test performances, the absence of such associations was not surprising considering the large heterogeneity in the prevalence of a cognitive deficit phenotype, which would, therefore, require a markedly larger sample size than the present 25 to enable the detection of such correlation. Neuroimaging studies in particular benefit from greater sample sizes (34). Another consideration is that some cortical structures showed multiple subregions affected; in terms of whether one cortical structure can have meaningfully distinct subregions, we have shown that for one area, the insular cortex, that subregions are functionally distinct (72). Thus, within one structure, the different subregions affected in pediatric OSA may have different neuropsychological consequences. Furthermore, cortical thickness changes may reflect a late-stage of brain alterations (since atrophy occurs over many years), and other measures of structure may detect earlier pathology. Since OSA can go for years undetected, it is unclear how long the participants in our study had the disorder, or how long they neuropsychological performance may have been affected. For example, within the hippocampus, changes in structure measured by water diffusivity are correlated with neuropsychological function (37). Whether such subtle structural changes extend to the cortex is unclear, but assessment of water diffusion specifically within the cortex is warranted for future studies.

In summary, we present evidence of regionally defined cortical thinning and thickening in children with OSA compared to healthy controls. These findings clearly indicate the presence of injury to the brain that reflects either the consequences of neuronal cell losses, the imposition of perturbed maturation brain processes, or a combination thereof in those sites with cortical thinning and hypoxia-induced inflammatory changes in sites with cortical thickening. Further studies evaluating the effect of intervention on these findings are needed.

## Ethics Statement

The study was approved by the human ethics committee at the University of Chicago (IRB Protocol # 11-0280-CR004), and written informed consent was obtained from the legal caregiver of each participant in accordance with the Declaration of Helsinki. Assent was obtained from children >7 years old.

## Author Contributions

PM performed major components of image data analysis and contributed to manuscript editing. LK-G conceptualized the study, recruited subjects, coordinated the database, analyzed data, and drafted portions of the manuscript. JP performed data analyses. RM performed data analyses. RK performed components of data analyses and interpretation and contributed to manuscript editing. MP performed sleep study scoring and interpretation, coordinated and performed the MRI scans. DG participated in the conceptualizing the study and edited the manuscript.

## Conflict of Interest Statement

The authors declare that the research was conducted in the absence of any commercial or financial relationships that could be construed as a potential conflict of interest.

## Footnotes

^1^http://web.ispad.org/sites/default/files/resources/files/idf-ispad_diabetes_in_childhood_and_adolescence_guidelines_2011_0.pdf

^2^http://stokes.chop.edu/web/zscore.

^3^www.nhlbi.nih.gov/guidelines/hypertension/child_tbl.htm.

^4^https://pediatricmri.nih.gov/nihpd/info/index.html.
